# Identification of Candidate Biomarkers Correlated With the Pathogenesis and Prognosis of Non-small Cell Lung Cancer via Integrated Bioinformatics Analysis

**DOI:** 10.3389/fgene.2018.00469

**Published:** 2018-10-12

**Authors:** Mengwei Ni, Xinkui Liu, Jiarui Wu, Dan Zhang, Jinhui Tian, Ting Wang, Shuyu Liu, Ziqi Meng, Kaihuan Wang, Xiaojiao Duan, Wei Zhou, Xiaomeng Zhang

**Affiliations:** ^1^Department of Clinical Chinese Pharmacy, School of Chinese Materia Medica, Beijing University of Chinese Medicine, Beijing, China; ^2^Evidence Based Medicine Center, School of Basic Medical Sciences, Lanzhou University, Lanzhou, China; ^3^Key Laboratory of Evidence Based Medicine and Knowledge Translation of Gansu Province, Lanzhou, China; ^4^Beijing Institute of Traditional Chinese Medicine, Beijing University of Chinese Medicine, Beijing, China

**Keywords:** non-small cell lung cancer, bioinformatics, differentially expressed genes, survival, biomarker, GEO

## Abstract

**Background and Objective:** Non-small cell lung cancer (NSCLC) accounts for 80–85% of all patients with lung cancer and 5-year relative overall survival (OS) rate is less than 20%, so that identifying novel diagnostic and prognostic biomarkers is urgently demanded. The present study attempted to identify potential key genes associated with the pathogenesis and prognosis of NSCLC.

**Methods:** Four GEO datasets (GSE18842, GSE19804, GSE43458, and GSE62113) were obtained from the Gene Expression Omnibus (GEO) database. The differentially expressed genes (DEGs) between NSCLC samples and normal ones were analyzed using limma package, and RobustRankAggreg (RRA) package was used to conduct gene integration. Moreover, Search Tool for the Retrieval of Interacting Genes database (STRING), Cytoscape, and Molecular Complex Detection (MCODE) were utilized to establish protein–protein interaction (PPI) network of these DEGs. Furthermore, functional enrichment and pathway enrichment analyses for DEGs were performed by Funrich and OmicShare. While the expressions and prognostic values of top genes were carried out through Gene Expression Profiling Interactive Analysis (GEPIA) and Kaplan Meier-plotter (KM) online dataset.

**Results:** A total of 249 DEGs (113 upregulated and 136 downregulated) were identified after gene integration. Moreover, the PPI network was established with 166 nodes and 1784 protein pairs. Topoisomerase II alpha *(TOP2A)*, a top gene and hub node with higher node degrees in module 1, was significantly enriched in mitotic cell cycle pathway. In addition, Interleukin-6 *(IL-6*) was enriched in amb2 integrin signaling pathway. The mitotic cell cycle was the most significant pathway in module 1 with the highest *P*-value. Besides, five hub genes with high degree of connectivity were selected, including *TOP2A, CCNB1, CCNA2, UBE2C*, and *KIF20A*, and they were all correlated with worse OS in NSCLC. **Conclusion:** The results showed that *TOP2A, CCNB1, CCNA2, UBE2C, KIF20A*, and *IL-6* may be potential key genes, while the mitotic cell cycle pathway may be a potential pathway contribute to progression in NSCLC. Further, it could be used as a new biomarker for diagnosis and to direct the synthesis medicine of NSCLC.

## Introduction

Lung cancer is the crucial cause of cancer-related mortality in China and worldwide. In 2016, the number of patients newly diagnosed with lung cancer will be 224 000, and over 158 000 will die from it in the United States alone (Torre et al., 2016; Sperduto et al., 2017). Non-small cell lung cancer (NSCLC) accounts for 80–85% of all patients with lung cancer, which is also the most malignant carcinoma among men and women, with an incidence higher than the combined incidence of breast, cervical, and colorectal cancers (Spiro and Porter, 2002; Maher et al., 2012). Although prominent progress in early diagnosis and treatment methods, 5-year relative overall survival (OS) rate is less than 20% (Boolell et al., 2015; Lin et al., 2018). For inoperable cancer patients and surgical patients chemotherapy remains the most important complementary treatment, and platinum is mild in the treatment of advanced NSCLC (Song et al., 2014). However, the adverse drug reactions are getting worse and drug resistance has also been emerging. Therefore, the novel strategies are urgently needed to supplement traditional chemotherapy (Lu et al., 2018). Over the past decades, our understanding of the molecular characterization of cancer has increased though genomic medicine. The treatment strategy for advanced NSCLC has changed from the traditional chemotherapy based on histopathology to individualized precision treatment based on carcinogenic factors (Jin et al., 2018). Zhu et al indicated that MTA1 might be a momentous biomarker in the diagnosis of NSCLC. (Zhu et al., 2017) Some studies revealed that high expression of IGF-1R and loss of PTEN were associated with poor prognosis in NSCLC. (Zhao J. et al., 2017; Zhao Y. et al., 2017) Although biomarkers and therapeutic targets found in NSCLC have made a great contribution to improving the diagnosis and treatment of NSCLC, due to the biological complexity and poor prognosis of NSCLC, more genetic information remains urgently needed to provide reference for precision medical treatment (Riess et al., 2018; Xu et al., 2018).

In order to explore common biomarkers associated with cancer and direct drugs for cancer treatment, diagnosis and prognosis, more and more microarray and high throughput sequencing technologies on cancer have been released in recent years (Kulasingam and Diamandis, 2008; Matamala et al., 2015; Lusito et al., 2018; Zhang et al., 2018). Meanwhile, in order to overcome the limited or inconsistent results caused by the application of different technological platforms or a small sample size, integrated bioinformatics methods have been applied in cancer research and a vast range of valuable biological information has been uncovered (Chang et al., 2015; Lee et al., 2015; Sun M. et al., 2017; Li et al., 2018).

**FIGURE 1:**
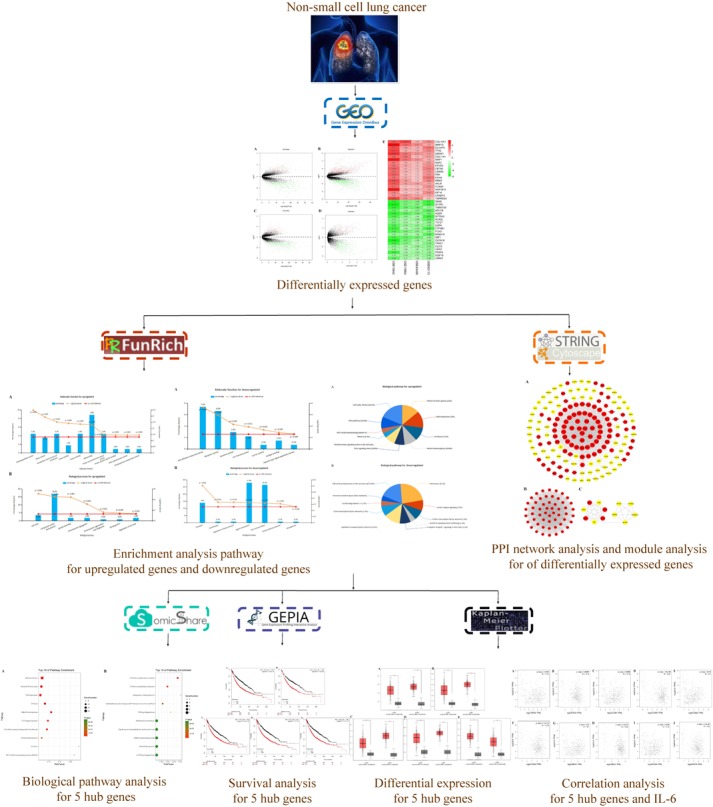
Workflow for identification of core genes and pathways for non-small cell lung cancer.

The microarray data of GSE18842 (Sanchez-Palencia et al., 2011), GSE19804 (Lu et al., 2010), GSE43458 (Kabbout et al., 2013), and GSE62113 (Li et al., 2014) were applied to identify the differentially expressed genes (DEGs) between NSCLC tissues and normal ones utilizing a bioinformatics approach. In addition, a protein–protein interaction (PPI) network of 166 hub genes and two modules was established. Meanwhile, five significant genes were found to be associated with OS in NSCLC via Kaplan Meier-plotter online database. Besides, enrichment analyses were performed for DEGs. The present study aimed to identify key genes associated with the pathogenesis and prognosis of NSCLC from new insights. The workflow of this study was shown in **Figure 1**.

## Materials and Methods

### Gene Expression Profile Data

The gene expression profile data (GSE18842, GSE19804, GSE43458, and GSE62113) were obtained from Gene Expression Omnibus (GEO^1^) (Schminke et al., 2015). All included datasets met the following criteria: (1) they employed tissue samples gathered from human NSCLC and corresponding adjacent or normal tissues. (2) they included at least 10 samples. (3) all the studies on these datasets were published in English language.

### Integrated Analysis of Microarray Datasets

Limma package (Ritchie et al., 2015) in R/Bioconductor software was applied to perform the normalization and log_2_ conversion for the matrix data of each GEO dataset, and the DEGs in every microarray were also screened by the limma package. Gene integration for the DEGs identified from the four datasets was conducted employing RobustRankAggreg (Kolde et al., 2012). | log_2_FC|≥ 1 and adjust *P-*value < 0.05 were considered statistically significant for the DEGs.

### Functional Enrichment Analysis

FunRich is a stand-alone software tool used mainly for functional enrichment and interaction network analysis of genes and proteins (Pathan et al., 2017). The functional enrichment analysis for the upregulated and downregulated DEGs, which included molecular function (MF), biological process (BP), cellular component (CC), and biological pathway (BPA), was performed via FunRich in the present study. The results of the functional enrichment analysis were visualized via OmicShare platform^2^ (OmicShare, 2018).

### PPI Network and Module Analysis

The Search Tool for the Retrieval of Interacting Genes database (STRING)^3^ provides information regarding the predicted and experimental interactions of proteins (Szklarczyk et al., 2015). In the present study, the DEGs were mapped into PPIs and a combined score of ≥0.4 was used as the cut-off value. Moreover, the use of Cytoscape software (version 3.6.0) was to construct PPI networks (Shannon et al., 2003). The network module was one of the characteristics of the protein network and may contain specific biological significance. The Cytoscape plug-in Molecular Complex Detection (MCODE) was applied to detect notable modules in this PPI network (Bader and Hogue, 2003). Degree cutoff = 2, Node Score Cutoff = 0.2, and *K*-Core = 2 were set as the advanced options. Next, the enrichment analysis of the DEGs in different modules was also conducted by the Funrich software.

### Survival Analysis of Hub Genes

Kaplan Meier-plotter (KM plotter^4^) could assess the effect of 54675 genes on survival using 10,461 cancer samples (Lánczky et al., 2016; Szász et al., 2016). The aim is to estimate the time of death, an event that will eventually occur in each person, which may have important effects when using these estimates to inform clinical decisions, health care policies and resource allocation (Lacny et al., 2018). The relapse free and OS information were based on GEO (Affymetrix microarrays only), EGA and TCGA database. The hazard ratio (HR) with 95% confidence intervals and log rank *P-*value were calculated and showed on the plot (Sun C. et al., 2017).

### Expression Level Analysis and Correlation Analysis of the Hub Genes

The Gene Expression Profiling Interactive Analysis (GEPIA)^5^ is a newly web-based tool for gene expression analysis between the tumor and normal data from the Cancer Genome Atlas (TCGA) and the Genotype-Tissue Expression (GTEx), applying a standard processing pipeline (Tang et al., 2017). It provides customizable functions such as tumor and normal differential expression analysis, and we could demonstrate the expression of hub genes in NSCLC tissues and normal ones. Then the boxplot was performed to visualize the relationship (Sun C. et al., 2017). Correlation analysis performs pairwise gene correlation analysis for any given sets of TCGA and/or GTEx expression data and check the relative ratios between two genes (Tang et al., 2017).

## Results

### Gene Expression Profile Data

**FIGURE 2:**
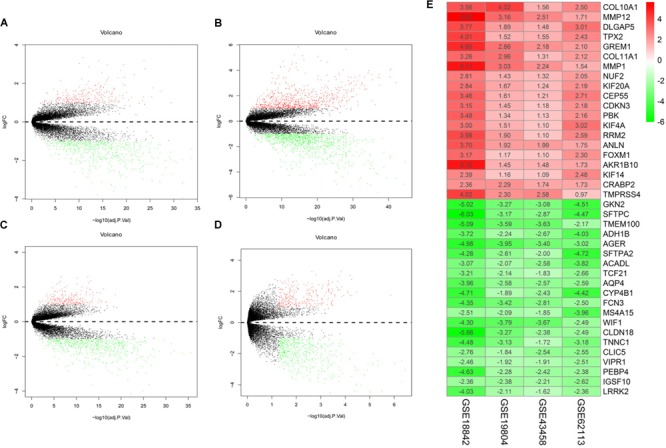
Volcano plot of gene expression profile data in NSCLC samples and normal ones and heat map of differentially expressed gene (DEGs). **(A)** Volcano plot of GSE19804, **(B)** volcano plot of GSE18842, **(C)** volcano plot of GSE43458, **(D)** volcano plot of GSE62113, and **(E)** heat map of differentially expressed genes. Green represents a lower expression level, red represents higher expression levels and white represents that there is no different expression amongst the genes. Each column represents one dataset and each row represents one gene. The number in each rectangle represents the normalized gene expression level. The gradual color ranged from green to red represents the changing process from down-regulation to up-regulation.

There were 197 NSCLC samples and 154 normal samples in this study (**Table 1** and **Supplementary Table S1**). In all, 249 genes (113 upregulated and 136 downregulated genes) were identified as DEGs in the NSCLC samples compared with the normal ones (**Figures 2A–D** and **Supplementary Table S2**). According to the cut-off criteria, we screened the top 20 differentially expressed upregulated and downregulated genes (**Figure 2E**).

**Table 1:** The gene expression profile data characteristics.

Reference	PMID	Record	Tissue	Platform	Normal	Tumor
Lu et al., 2010	20802022	GSE19804	NSCLC	GPL570- [HG-U133_Plus_2] Affymetrix Human Genome U133 Plus 2.0 Array	60	60
Sanchez-Palencia et al., 2011	20878980	GSE18842	NSCLC	GPL570-[HG-U133_Plus_2] Affymetrix Human Genome U133 Plus 2.0 Array	45	46
Kabbout et al., 2013	23659968	GSE43458	NSCLC	GPL6244- [HuGene-1_0-st] Affymetrix Human Gene 1.0 ST Array [transcript (gene) version]	30	80
Li et al., 2014	25429762	GSE62113	NSCLC	GPL14951- Illumina HumanHT-12 WG-DASL V4.0 R2 expression beadchip	19	11

### Enrichment Analyses

**FIGURE 3:**
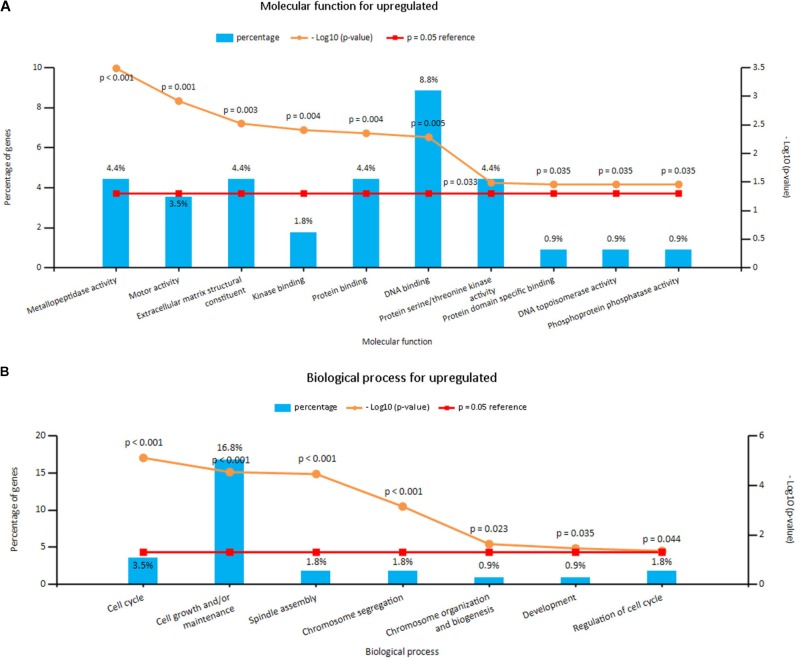
**(A)** Molecular function for upregulated genes, **(B)** biological process for upregulated genes. *X* axis represents molecular functions or biological processes; *Y* axis represents percentage of genes or –log10(*P*-value).

**FIGURE 4:**
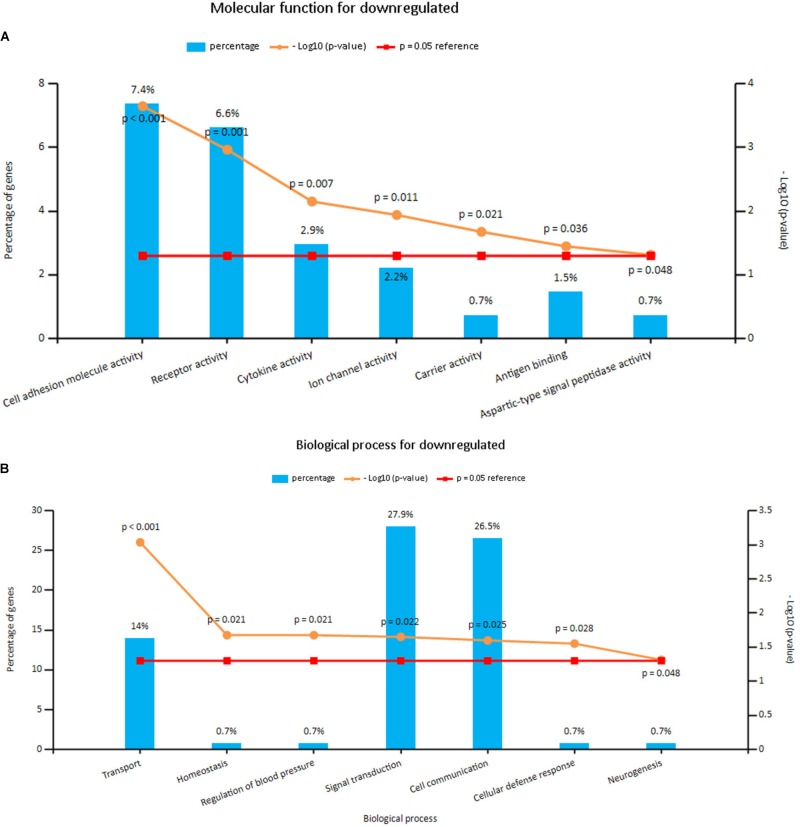
**(A)** Molecular function for downregulated genes, **(B)** biological process for downregulated genes. *X* axis represents molecular functions or biological processes; *Y* axis represents percentage of genes or –log10(*P*-value).

Enrichment analyses for the upregulated and downregulated DEGs after gene integration were performed via Funrich. The functional enrichment terms of upregulated DEGs were mainly associated with the metallopeptidase activity, cell communication and cell growth and/or maintenance (**Figures 3A,B** and **Supplementary Table S3**). The downregulated DEGs were mainly enriched in the cell adhesion molecule activity, receptor activity and transport (**Figures 4A,B** and **Supplementary Table S3**).

**FIGURE 5:**
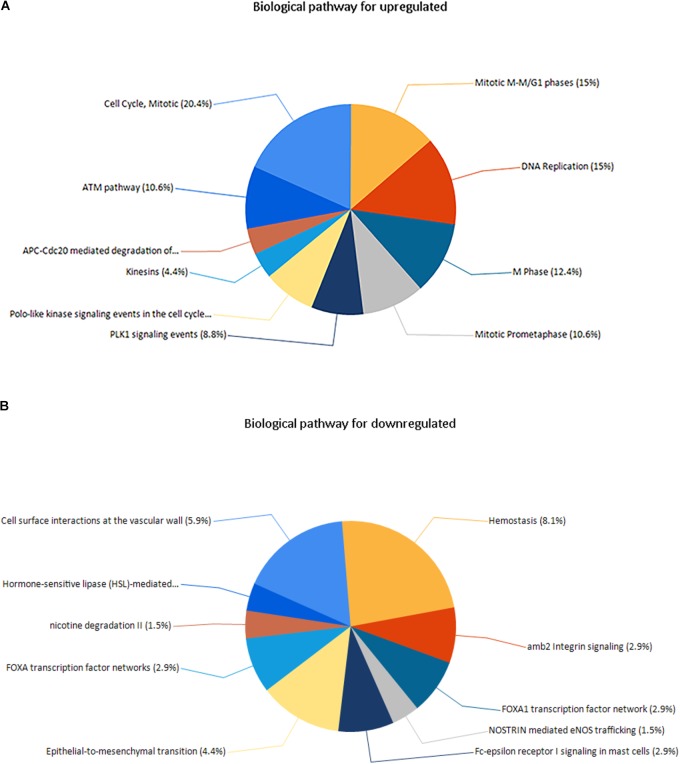
**(A)** Biological pathway for upregulated genes, **(B)** biological pathway for downregulated genes.

Three pathways that were particularly enriched by upregulated DEGs were mitotic cell cycle, DNA replication and mitotic M-M/G1 phases. Furthermore, a critical gene *TOP2A* was significantly enriched in mitotic cell cycle pathway, validated transcriptional targets of deltaNp63 isoforms pathway, p63 transcription factor network pathway, mitotic G1-G1/S phases pathway and G0 and early G1 pathway in biological pathway (BPA) enrichment analysis for upregulated genes. (**Figure 5A** and **Supplementary Table S3**).

Downregulated DEGs were notably enriched in two pathways, including Hemostasis, cell surface interactions at the vascular wall and amb2 integrin signaling. However, a vital gene Interleukin-6 (*IL-6*) was significantly enriched in amb2 integrin signaling pathway, integrin family cell surface interactions pathway, glypican pathway and glucocorticoid receptor regulatory network pathway in BPA enrichment analysis for downregulated genes (**Figure 5B** and **Supplementary Table S3**).

### PPI Network Analysis and Module Analysis

**FIGURE 6:**
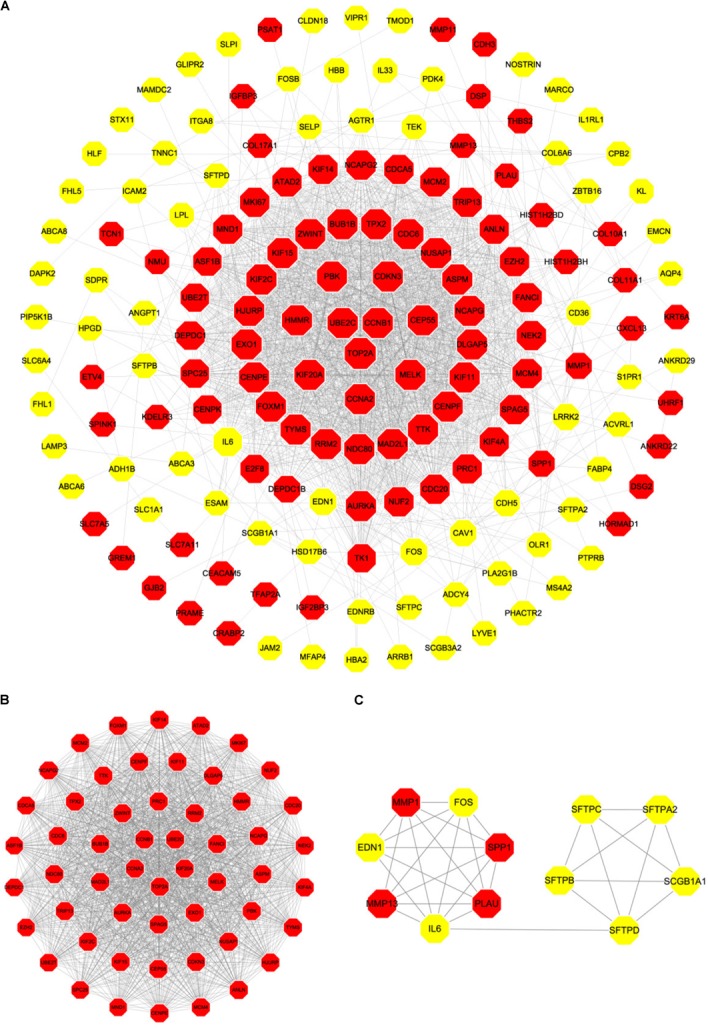
PPI network of differentially expressed genes in NSCLC samples compared with the control ones and two significant modules identified from the PPI network using the molecular complex detection method with a score of >5.0. Red nodes, upregulated genes; Yellow nodes, downregulated genes; **(A)** PPI network of differentially expressed genes in NSCLC samples compared with the control ones; **(B)** Module 1, MCODE score = 52.34; **(C)** Module 2, MCODE score = 5.63.

**FIGURE 7:**
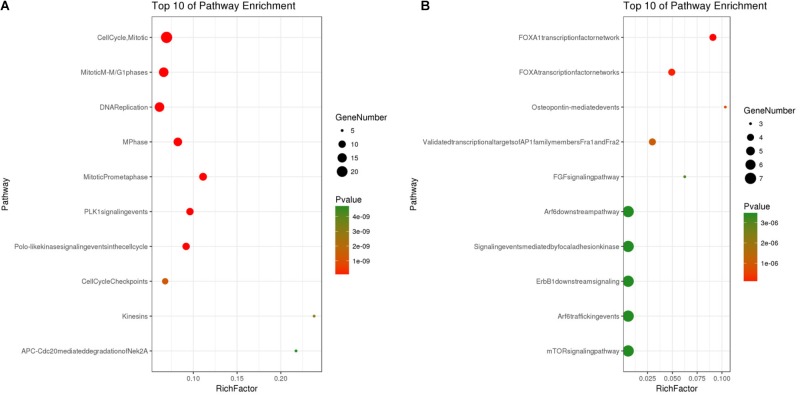
**(A)** Pathway analysis of Module 1; **(B)** Pathway analysis of Module 2. The *y*-axis shows significantly enriched pathways of Module 1 and Module 2, and the *x*-axis shows the Rich factor, *P* < 0.01, FDR < 0.01. Rich factor stands for the ratio of the number of target genes belonging to a pathway to the number of all the annotated genes located in the pathway. The higher Rich factor represents the higher level of enrichment. The size of the dot indicates the number of target genes in the pathway, and the color of the dot reflects the different *P*-value range.

Based on the SRTING database, we made the PPI network of a total of 166 nodes and 1784 protein pairs were obtained with a combined score >0.4. As shown in **Figure 6A** and **Supplementary Table S4**, the majority of the nodes in the network were upregulated DEGs in NSCLC samples. In total, two modules (modules 1 and 2) with score >5 were detected by MCODE. As shown in **Figures 6B,C**, TOP2A, CCNB1, CCNA2, UBE2C, and KIF20A were hub nodes with higher node degrees in module 1, and IL-6, MMP1, SPP1, FOS, PLAU, EDN1, MMP13, and SFTPD were hub nodes in module 2. Besides, 5 hub genes with high degree of connectivity were selected (**Table 2**). Furthermore, module 1 and module 2 enrichment pathways were shown in **Figure 7** and **Supplementary Table S5**, the mitotic cell cycle pathway was identified as the most significant pathway in module 1.

**Table 2:** Hub genes with high degree of connectivity.

Gene	Degree	type	MCODE Cluster
*TOP2A*	69	up	Cluster 1
*CCNB1*	60	up	Cluster 1
*CCNA2*	59	up	Cluster 1
*UBE2C*	59	up	Cluster 1
*KIF20A*	58	up	Cluster 1

### The Kaplan Meier-Plotter and Expression Level of Hub Genes Correlation and Correlated Analysis

**FIGURE 8:**
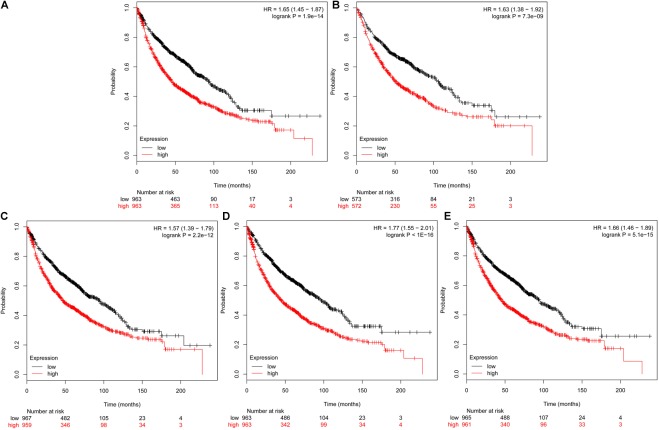
Prognostic roles of five hub genes in the NSCLC patients. Survival curves are plotted for NSCLC cancer patients. **(A)** TOP2A; **(B)** CCNB1; **(C)** CCNA2; **(D)** UBE2C; and **(E)** KIF20A.

**FIGURE 9:**
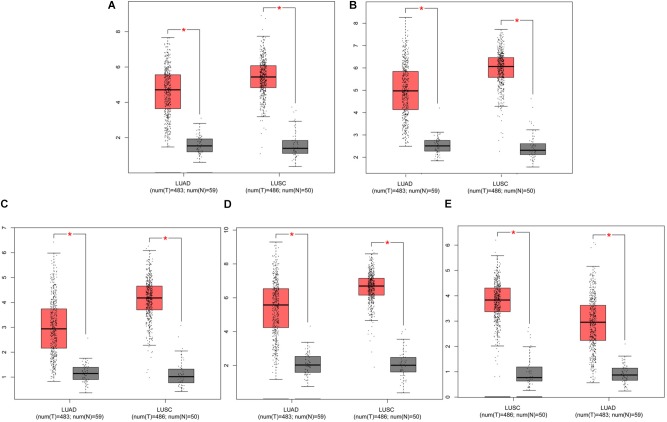
Analysis of five hub genes expression level in human NSCLC. The red and gray boxes represent cancer and normal tissues, respectively. **(A)** TOP2A; **(B)** CCNB1; **(C)** CCNA2; **(D)** UBE2C; and **(E)** KIF20A; LUAD: Lung adenocarcinoma; LUSC: Lung squamous cell carcinomas.

**FIGURE 10:**
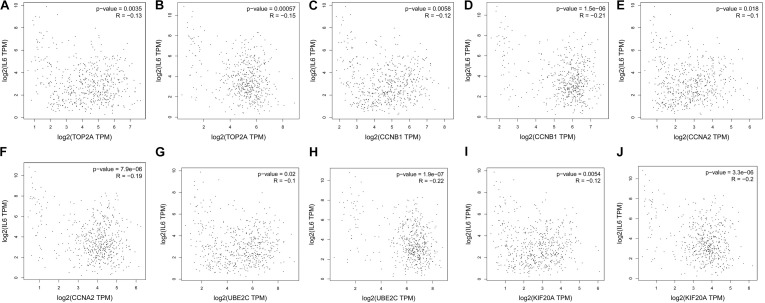
Correlation analysis of 5 hub genes and IL-6 in NSLCL. **(A,C,E,G,I)** lung adenocarcinoma; **(B,D,F,H,J)** lung squamous cell carcinomas.

The prognostic information of the 5 hub genes was freely available in Kaplan Meier-plotter. It was found that high expression of *TOP2A* [HR 1.65 (1.45–1.87), *P* = 1.3*e*–14], *CCNB1* [HR 1.63 (1.38–1.92), *P* = 7.3*e*–09], *CCNA2* [HR 1.57 (1.39–1.79), *P* = 2.2*e*–12)], *UBE2C* [HR 1.77 (1.55–2.01), *P* < 1*e*–16], and *KIF20A* [HR 1.66 (1.46–1.89), *P* = 5.1*e*–15] was associated with worse OS for NSCLC patients. (**Figure 8**) Then, we applied GEPIA to catch the hub genes expression level between NSCLC tissues and normal ones, and **Figure 9** reflected that compared with the normal ones, the 5 genes significantly increased expression levels in NSCLC tissues. The increase of 5 hub genes was interacted strongly with the decrease of *IL-6* which was observed in the LUAD (**Figures 10A,C,E,G,I**) and LUSD (**Figures 10B,D,F,H,J**).

## Discussion

In the present study, the gene expression patterns obtained from the GEO database revealed a total of 249 genes, including 113 upregulated and 136 downregulated genes, which were differently expressed in NSCLC samples compared with controls. The upregulated genes with *TOP2A* as a hub gene were significantly enriched in the mitotic cell cycle pathway. The downregulated genes with IL-6 as a hub gene were significant enriched in the amb2 integrin signaling pathway. Five hub genes *(TOP2, CCNB1, CCNA2, UBE2C*, and *KIF20A*) which were up-regulated in NSCLC tissues in comparison to normal tissues. Meanwhile, increased of five hub genes was associated with worse OS and decrease of *IL-6*.

Type II topoisomerases contain two types of isozymes: *TOP2A* and topoisomerase II beta (*TOP2B*) (Li and Liu, 2001; Chen et al., 2012). High expression of *TOP2A* is detected in several types of cancer, and more importantly *TOP2A* has been acknowledged as a cancer target in clinical application (Wesierska-Gadek and Skladanowski, 2012; Lan et al., 2014; Li et al., 2015). In many tumors, such as breast cancer, head, and neck squamous cell carcinoma and NSCLC, *TOP2A* expression is significantly higher in middle and low differentiated tumors than in high differentiated ones (Nakopoulou et al., 2000; Stathopoulos et al., 2000; Sun and Wu, 2004). Highly increased expression level of *TOP2A* in NSCLC tissues is closely related to the malignant biological behaviors of this cancer such as proliferation and invasion, and interference with *TOP2A* expression inhibits the proliferation and invasion of NSCLC cells (Han et al., 2016). Higher *TOP2A* expression in NSCLC predicts more malignant biological behavior of the tumor, and more importantly *TOP2A* has been widely used as an independent prognostic factor in NSCLC and high expression of *TOP2A* is associated with worse prognoses of NSCLC patients (Villman et al., 2002). In the present study, *TOP2A*, a hub node with higher node degree in module PPI network, was enriched in mitotic cell cycle pathway and validated transcriptional targets of deltaNp63 isoforms pathway. Therefore, the results are in line with these previous studies, which indicated that *TOP2A* may be directly or indirectly important in NSCLC development and worse OS.

Interleukin-6 is a key cytokine, which involves in various pathological and physiological processes of inflammatory reaction and proliferation and differentiation of various malignant tumor cells (Kishimoto, 2005; Hong et al., 2007; Ando et al., 2014). *IL-6* has been reported to be critical in the tumorigenesis and tumor metastasis of epithelial cancer (Shintani et al., 2016). The unbalanced of *IL-6* and its receptors will affect the stability of the internal environment of the body, which will also lead to immune dysfunction and induce the occurrence and development of tumors (Xu et al., 2002). Previous studies have shown that *IL-6* is a potential target for the treatment of patients with advanced NSCLC. Moreover, higher levels of *IL-6* exists in NSCLC patients and shows an upward trend and *IL-6* is associated with the pathogenesis and progression of lung cancer (Strassmann et al., 1992; Xu et al., 2002; Chang et al., 2013). However, some evidence showed that *IL-6* is down-regulated in NSCLC (Fang et al., 2017). The inconsistent results of the present studies in turn show that *IL-6* may be play an important role in NSCLC development. The fact validates our results, which identified *IL-6* as a hub gene.

In addition to the two aforementioned genes, *NDC80, CCNA2, CDC6, CCNB1, TPX2, AURKA, MAD2L1*, and *BUB1B* are enriched in mitotic cell cycle pathway, which is the most highly enriched pathway of module 1 with the most significant *P*-value. For cancer screening and prognosis, analysis of the DNA replication initiation machinery and mitotic engine proteins in human tissues is now conducive to the identification of novel biomarkers and is suppling target validation for cell cycle-directed therapies (Williams and Stoeber, 2011). Therefore, the mitotic cell cycle pathway and its mentioned genes may be vital in NSLCL progression.

Cyclin B1 (*CCNB1*) is a regulatory protein, which plays a crucial role in mitosis. Overexpressed *CCNB1* was detected in NSCLC and related to the clinic stages of NSCLC, and could be used as a marker for NSCLC in indicating the abilities of division, proliferation and apoptosis inhibition of NSCLC (Li et al., 2011). Cyclin A2 (*CCNA2*) is one of the mammalian A-type cyclin family in humans (Ko et al., 2013). Several research teams have reported the prognostic significance of *CCNA2* in lung cancer but the results are controversial. Some suggested that the expression *CCNA2* is negatively correlated with prognosis (Volm et al., 1997; Dobashi et al., 2003). However, others reported *CCNA2* could not serve as a prognostic factor (Müllertidow et al., 2001; Cooper et al., 2010). Ubiquitin-conjugating enzyme E2C (*UBE2C*), which encodes a member of the E2 ubiquitin-conjugating enzyme family, had been reported to serve momentous roles in various malignancies, including breast cancer, colorectal cancer, and hepatocellular carcinoma (Ieta et al., 2007; Loussouarn et al., 2009; Chen et al., 2010; Bavi et al., 2011). For lung cancer, a study showed that progression-free survival and poorer OS of NSCLC patients was associated with *UBE2C* overexpression (Kadara et al., 2009; Zhang et al., 2015). Kinesin family member 20A (*KIF20A*) belongs to the kinesin superfamily-6, a microtubule-correlated motor protein, is required for cell cycle mitosis (Yan et al., 2012; Zhang et al., 2014). Based on previous studies, *KIF20A* has been overexpressed in lung and breast cancer, otherwise low levels are inspected in the placenta and heart (Lai et al., 2000; Kikuchi et al., 2003; Stangel et al., 2015). Concerning malignant cellular functions, *KIF20A* has been revealed to be involved in proliferation, migration, invasiveness, and angiogenesis (Taniuchi et al., 2014).

At present, some relevant studies were published that concerned about core genes in NSCLC in the database. Huang et al identified five genes from two GEO datasets by developing an integrated method including the raw data analysis by GEO2R, functional and pathway enrichment analysis, PPI network and module analysis, cell culture, reverse transcription-quantitative polymerase chain reaction, ROC analysis, survival analysis of hub genes, and statistical analysis (Huang and Gao, 2018). Piao et al identified 16 hub genes, the expression of 14 of which were associated with prognosis of NSCLC patients by a bioinformatics approach incorporating functional and pathway enrichment analysis, PPI network and OS analysis based on gene and miRNA expression profiles from the GEO database (Piao et al., 2018). Chen et al identified 8 disease genes from one GEO database by using Naïve Bayesian Classifier based on the Maximum Relevance Minimum Redundancy feature selection method following preprocessing, shortest path analysis and function and pathway enrichment analysis (Chen et al., 2018). Tian et al identified 7 important genes from one GEO database by using data preprocessing and screening of DEGs, functional enrichment analysis and construction of transcriptional regulatory network (Tian et al., 2016). Compared to previous works, the advantages of the current study were mainly reflected in the following points: First, this study integrated microarray data with relative large sample size from multiple GEO datasets. Secondly, functional enrichment analysis was further carried out to analyze the main biological functions modulated by the DEGs. Finally, this study built gene networks and identified potential diagnostic and prognostic biomarkers in NSCLC and the correlations between hub genes.

The limitations of our study were as follows: First, our results cannot be validated due to the absence of experiment. Second, the data used in our study were accessed from a public database while the quality of the data cannot be appraised. Third, the sample size of involved data was relatively small, and the study failed to cover different races and regions, which can affect the gene expression in NSCLC. Finally, as a result of our study only focused on the genes that are usually identified as significant changes in multiple data sets, there is no consideration of such characteristics as sex, age, tumor classification, and staging in detail. Thus, some biological information may be overlooked in our study.

## Conclusion

Our bioinformatics analysis identified *TOP2A, CCNB1, CCNA2, UBE2C, KIF20A*, and *IL-6* and the mitotic cell cycle pathway may be critical in the development and prognosis of NSCLC. However, further experiments confirming the results of this prediction in NSLCL are required because our study was performed based on data analysis. We hope this study may provide some evidence for the future genomic individualized treatment of NSCLC from new insights.

## Author Contributions

MN and XL conceived, designed, and performed the research and wrote the paper. JW supervised the research. JT provisioned useful suggestions in methodology. DZ, TW, SL, ZM, KW, XD, WZ, and XZ provisioned suggestions in figure preparation. All authors read and approved the final version of the manuscript.

## Conflict of Interest Statement

The authors declare that the research was conducted in the absence of any commercial or financial relationships that could be construed as a potential conflict of interest.

## Footnotes

1 http://www.ncbi.nlm.nih.gov/geo/

2 http://www.omicshare.com/ (accessed July 19, 2018)

3 https://string-db.org/

4 http://kmplot.com/analysis/

5 http://gepia.cancer-pku.cn/index.html
